# Important Parameter Groups in Thermal Protection of Polymers

**DOI:** 10.3390/ma8084679

**Published:** 2015-07-24

**Authors:** John Staggs

**Affiliations:** Energy Research Institute, School of Chemical and Process Engineering, University of Leeds, Leeds LS2 9JT, UK; E-Mail: j.e.j.staggs@Leeds.ac.uk; Tel.: +44-(0)-113-343-2495

**Keywords:** thermal protection, modelling, heat sink, intumescence

## Abstract

The problem of thermal protection is explored for two idiosyncratic reactive systems, namely a sacrificial heat-sink material and an intumescent system where a dynamically evolving insulation layer is produced from an initially thin coating. Relatively simple mathematical models of both systems are proposed that encompass the important physical characteristics of each situation and these models are analysed using a mixture of numerical and analytical techniques. Important dimensionless parameter groups are identified and domains of parameter space where thermal performance is particularly good- or particularly bad- are identified.

## 1. Introduction

Despite the advances in modelling various important methods of thermal protection, in particular reactive systems such as intumescent coatings or heat sink additives, there has been little investigation into the important engineering parameters that should be optimised for best performance. As a corollary to this, the parameters that underpin the basic dynamics of these systems have not been adequately explored in an engineering context. Most contributions to date have been either highly technical generalised mathematical analyses of reaction-diffusion systems or detailed engineering models of specific systems that do not look at overall behaviour. This contribution is oriented at bridging the gap between the two extremes. Stripped-back mathematical models that are simple enough to analyse in detail, yet still retain important physical details, are investigated. In particular, important parameter groups are identified for two important scenarios and their role in thermal protection is analysed. Both situations involve the use of a reactive component either as a heat sink or as a means of producing a porous insulation layer and, as we shall see, the analysis yields interesting insight into the significant dynamics for each case.

Before any analysis of thermal protection can be attempted, we must define what is meant by “best thermal performance”. Here, two commonly encountered situations are used to define optimality: minimisation of the temperature difference between an exposed surface and a vulnerable substrate for fixed heating conditions and maximisation of failure time (again for fixed heating conditions). Failure time in this context is defined as the time taken for a vulnerable substrate to reach a prescribed temperature. Naturally the rate at which heat is absorbed by a protecting layer depends on the nature of heat transfer from the external environment. In purely radiative heat exchange, the heat transfer rate can be minimised by the obvious device of minimising the surface absorptivity. For the purposes of this work the nature of the heat transfer from an external source is kept general so that in-depth properties, rather than surface properties, of the protecting layer are the main focus.

The discussion begins with a general overview of thermal insulation and the relationship between porosity and thermal performance is explored. Furthermore the role of heat transfer mechanisms other than conduction in porous materials is also discussed. Having established the general properties of inert layers, the dynamics of two reactive systems are then examined. Firstly a simple heat-sink system is analysed where a single-step in-depth endothermic reaction proceeds as the material is heated. Later, a simple dynamically evolving insulation layer is analysed where a reaction is assumed to occur in a thin coating that produces a thick insulating layer. In this case the rate of heat transfer to the unexposed surface is governed by a balance between the rate at which expansion occurs and the rate at which heat is added to the system.

## 2. Thermal Insulation

An obvious strategy for thermal protection of polymers involves the use of an insulating layer. The ultimate purpose of the insulation depends on the specific application. Two typical scenarios are:

■ Maximisation of the temperature difference between the exposed surface and the polymer.
■ Maximise the time for which the polymer remains below a specific temperature.

Such a layer could be inert or develop transiently via a reactive pathway (such as in the case of char-promoting systems). Consider for a moment an inert solid of thickness *L*, subject to a constant external heat flux *q"*. It may be shown that the difference between the temperature at the exposed surface and unexposed surface ∆*T* is given by

(1) $$\Delta T=\frac{{\dot{q}}^{\prime \prime }}{\lambda^{\prime }}\{\frac{1}{2}-\frac{2}{\pi^{2}}\sum_{n=1}^{\infty }e^{-n^{2}\pi^{2}\alpha t/L^{2}}\left(\frac{1-{\left(-1\right)}^{n}}{n^{2}}\right)\}.$$

Here *t* is time; α is thermal diffusivity and λ′ = λ/*L* is the conduction heat transfer coefficient, defined by the ratio of thermal conductivity λ to solid thickness *L*. It is clear from this expression that ∆*T* is a monotonic increasing function of time and that the maximum temperature difference is given by *q"*/(2λ′).

It follows that λ′ is a critical parameter for an insulating layer and in order to maximise ∆*T*, λ′ should be as small as possible. This implies that thermal conductivity should be as small as possible and the layer thickness as large as possible. Practical reasons usually restrict the thickness of the layer, so it is sensible to seek to minimise λ*.* Insulators are usually porous solids, comprising gas-filled pores embedded in a solid matrix. A simple argument shows that the composite thermal conductivity lies between two bounds, given by [1,2,3]

(2) $$\frac{\Lambda }{\Lambda +\phi \left(1-\Lambda \right)}\leq \frac{\lambda }{\lambda_{s}}\leq 1-\phi \left(1-\Lambda \right),$$

where Λ = λ*_g_*/λ*_s_*; φ is porosity (the ratio of pore volume to total volume) and the subscripts *s* and *g* refer to a solid (or *skeletal*) property and a pore property respectively. The maximum difference between the upper and lower bounds is λ*_s_*(1 − Λ^1/2^)^2^, which is at a porosity of Λ^1/2^/(1 + Λ^1/2^). For a given porosity, the actual value of thermal conductivity is dictated by the shape and orientation of the pores, the pore distribution and to a lesser extent, the pore size [2].

Correlations exist for a number of idealised situations featuring specifically shaped pores. Perhaps the most well known is Maxwell’s expression for spherical pores of modest porosity,

(3) $$\frac{\lambda }{\lambda_{s}}\approx \frac{2\phi (\Lambda -1)+\Lambda +2}{2+\Lambda -\phi (\Lambda -1)}$$

which is ~1 − 3φ/2 for small φ and Λ. Perhaps more realistic, but less convenient, is the implicit expression for randomly distributed spherical pores [4,5]:

(4) $$1-\phi =\frac{\left(\lambda /\lambda_{s}\right)-\Lambda }{{\left(\lambda /\lambda_{s}\right)}^{1/3}\left(1-\Lambda \right)}.$$

The theoretical thermal conductivity bounds suggest that increasing porosity will reduce thermal conductivity and hence heat transfer rate. If conduction was the only heat transfer mechanism occurring in a pore this would always be the case. However, there are two other modes of heat transfer that contribute to the total heat transfer rate across a pore: convection and radiation. If there is no gas flowing through the porous insulation, then free convection within a pore is the only possibility for a convective contribution. When the insulation layer is orientated such that temperature increases with vertical distance, there is a vacuum inside the pore or the environment is zero-gravity, then free convection is impossible. If this is not the case then the Rayleigh-Bénard instability triggers convection when the pore Rayleigh number *Ra* = *g*β∆*T_pore_*δ^3^/(α*_g_*ν*_g_*) reaches a critical value (typically of the order of 10^3^, but specific physical circumstances dictate the actual value). Here, *g* is gravitational acceleration; β is the expansion coefficient of the internal gas; ∆*T_pore_* is the temperature difference across the pore in the vertical direction; δ is the pore size and α*_g_*, ν*_g_* are the thermal diffusivity and kinematic viscosity of the internal gas respectively. For most practical applications, the Rayleigh-Bénard criterion suggests that pores of size less than ~1 cm will be too small to trigger internal convection.

Radiation through the interior region of a pore will also contribute to the total heat transfer, especially at high temperature. For small pores it transpires that this additional heat transfer may be accounted for approximately by augmenting the thermal conductivity [3,6], which to leading order produces an additional temperature-dependent term, giving the total thermal conductivity as λ*_tot_* = λ + φλ*_R_*(*T*^3^ − $T_{a}^{3}$)/$T_{a}^{3}$. Here λ*_R_* is a parameter that depends on pore size (together with other pore-specific variables, but not porosity) and *T_a_* is ambient temperature.

Now consider the case when a porous char layer is produced dynamically during thermal degradation of the original polymer. It is clear that porosity will change (probably increase) as time progresses. So it follows that the total thermal conductivity will be such that ∂λ*_tot_*/∂φ = ∂λ/∂φ + λ*_R_*(*T*^3^ − $T_{a}^{3}$)/$T_{a}^{3}$. In general λ will be a decreasing function of φ, implying that ∂λ/∂φ < 0, so a temperature T^*^ will exist such that when *T* < T^*^, λ*_eff_* will be a *reducing function* of φ and when *T* > T^*^, λ*_eff_* will be an *increasing function* of porosity. Clearly, the value of T^*^ will be important for the thermal effectiveness of the insulation: if T^*^ is less than the maximum temperature that the exposed surface will achieve, then the performance will be sub-optimal. From above, it is apparent that

(5) $$\frac{T^{*}}{T_{a}}={\left(1-\frac{1}{\lambda_{R}}\frac{\partial \lambda }{\partial \phi }\right)}^{1/3}\sim \frac{1}{\lambda_{R}^{1/3}}{\left(-\frac{\partial \lambda }{\partial \phi }\right)}^{1/3},$$

since in practice it is likely that λ*_R_* << 1. Note that the last relation becomes invalid if −∂λ/∂φ is very small, which is only the case if λ is close to the theoretical lower limit. For chars with porosity dependence close to the theoretical upper limit, λ/λ*_s_* ~ 1 − φ(1 − Λ) and since in practice it is likely that λ*_g_* << λ*_s_*, it follows that T^*^/*T_a_* ~ $\lambda_{R}^{\text{−1/3}}$. For very small pores, it may be shown that [6] λ*_R_* ≈ 4εσ$T_{a}^{3}$δ, where ε is the emissivity of the internal surface of the pore and σ is the Stefan-Boltzmann constant. In order to minimise λ*_tot_* we require λ*_R_* to be as small as possible, which implies that the pore size δ must be as small as possible (this also ensures that T^*^ is as large as possible). Hence an optimal porous insulator naturally has large porosity, but must consist of a large number of small pores, rather than a small number of large pores.

Now consider the requirement that the insulation must limit the temperature at the unexposed face for as long as possible, rather than maximising the temperature drop across its thickness. It transpires that the functional dependence of thermal conductivity on porosity plays a critical role in the performance of the insulating layer. The purpose of the insulating layer is now to minimise the heat transfer rate at the unexposed surface and this implies that the thermal diffusion timescale *t_D_* = *L*^2^/*a* must be as large as possible. The density and specific heat capacity of the insulation are ρ = (1 − φ)ρ*_s_* + φρ*_g_* ≈ (1 − φ)ρ*_s_* and *c* = (1 − φρ*_g_*/ρ)*c_s_* + φρ*_g_c_g_*/ρ ≈ *c_s_* respectively. Hence

(6) $$\frac{t_{D}}{\rho_{s}c_{s}L^{2}}\sim \frac{1-\phi }{\lambda }.$$

This last relation implies that, for a porous material, in order to maximise failure time $\tilde{\lambda }=\lambda /\left(1-\phi \right)$ should be as small as possible.

The question naturally arises that for what type of material is $\tilde{\lambda }$ a reducing function of porosity? In other terms, when is $d\tilde{\lambda }/d\phi <0$? If we look for example at the thermal conductivity upper bound λ/λ*_s_* = 1 − φ(1 − Λ) and ignore radiation, we find that

(7) $$\frac{d\tilde{\lambda }}{d\phi }=\Lambda /{\left(1-\phi \right)}^{2},$$

which clearly is always positive. If radiation is included at a fixed temperature, then from above, since increasing porosity increases the radiative contribution to λ*_tot_*, it follows that for the thermal conductivity upper bound ${\tilde{\lambda }}_{tot}$ will still always be an increasing function of φ. Hence, any insulation material whose functional dependence of thermal conductivity on porosity is close to the upper bound would not make a good choice in this context since increasing porosity for such a material would actually reduce the thermal protection.

It is interesting that if ignition resistance is the goal, *i.e.*, maximising the exposure time prior to ignition, then the low thermal conduction coefficient strategy is counter-productive. The reason for this is that if thermal conductivity is low, then heat transfer away from the exposed surface is also low. This means that material away from the exposed surface remains at a low temperature but most of the absorbed heat is confined to a region close to the exposed surface, implying that the temperature of the exposed surface increases rapidly. Degradation reactions in this region therefore proceed quickly and the rate of production of combustible volatile species is high, resulting in rapid ignition.

Many models of ignition have been considered in the literature. Traditional engineering approaches have applied simplifying criteria such as the assumptions that ignition occurs at a critical surface temperature or at a critical mass flux of combustible volatile gases [7,8,9,10,11,12,13,14,15,16,17]. The simplest of these is the critical surface temperature assumption, since the ignition problem then reduces to a straightforward heat transfer calculation. Looking at this behaviour in detail for thick specimens, consider the dimensionless diffusion equation, θ_τ_ = θ*_xx_*, where θ = (*T* − *T_a_*)T^*^, T^*^ being some appropriate temperature scale (to be specified below), τ is the ratio of time to diffusion time scale and *x* is the ratio of distance from the exposed surface to thickness. Integrating this equation over thickness, gives the ordinary differential equation ${\bar{\theta }}_{\tau }={\left[\theta_{x}\right]}_{x=0}^{x=1}$, where $\bar{\theta }\left(\tau \right)=\int_{x=0}^{x=1}\theta \left(x,\tau \right)dx$ is the average temperature in the sample. Assuming that the sample is well insulated on the unexposed surface, so that ${\theta_{x}|}_{x=1}=0$, we have that ${\bar{\theta }}_{\tau }=-{\theta_{x}|}_{x=0}$. For simplicity, if we ignore heat losses (which will be reasonable for the case of very high incident heat flux) then $-{\theta_{x}|}_{x=0}={\dot{q}}^{\prime \prime }/\left(T^{*}\lambda^{\prime }\right)$, where T* is the temperature scale. Thus, setting η = *q"*/(T^*^λ′) and denoting the surface temperature by θ_0_(τ), we have that in the region of *x* = 0, θ ~ θ_0_(τ) − η*x*, 0≤ *x* ≤ θ_0_/η, and θ ~ 0 for *x* > θ_0_/τ. Hence, the average temperature is such that $\bar{\theta }\sim \int_{0}^{\theta_{0}/\eta }\left(\theta_{0}-\eta x\right)dx=\theta_{0}^{2}/\left(2\eta \right)$ and so the surface temperature will vary with time approximately according to θ_0_(τ) ~ η(2τ)^1/2^. Hence, if it is assumed that ignition occurs at a characteristic surface temperature *T_ig_*, setting T^*^ = *T_ig_* − *T_a_*, we find that the time taken for ignition *t_ig_* is approximately:

(8) $$\frac{\alpha t_{ig}}{L^{2}}\sim \frac{1}{2}{\left(\frac{\lambda^{\prime }T_{ig}}{{\dot{q}}^{\prime \prime }}\right)}^{2}{\left(1-\frac{T_{a}}{T_{ig}}\right)}^{2}.$$

This last relationship is interesting for it suggests that ignition time has a functional dependence of the form *t_ig_* ~ *t_D_f* (λ′*T_ig_*/*q"*, *T_ig_*/*T_α_*), where *t_D_* is the diffusion time scale defined above. Furthermore, for fixed heating conditions, it follows that in order to maximise ignition resistance, the material parameter λ^2^/α = λρ*c*, known as thermal inertia, must be as large as possible. For a porous material, λρ*c* ~ λ(1 − φ)ρ*_s_c_s_* and so for optimal ignition resistance it follows that λ(1 − φ) should be as large as possible.

Recent contributions on ignition at high incident heat flux [18,19], have shown that for infra-red heat sources the ignition time—heat flux dependence predicted by Equation (8) above is reliable only for moderate heat fluxes up to ~40 kWm^−2^, particularly for PMMA. At higher heat flux, it appears that infra-red absorptivity has a significant effect on ignition time and that observed ignition resistance is actually greater than that predicted by the simple theory above.

## 3. Reactive Sacrificial Additives

The concept of a sacrificial coating for thermal protection is not new and has previously been implemented in heat shields for space vehicles [20]. The basic properties of ablative coatings have been summarised in [21]. Here the term *ablation* refers to the thermal degradation of a low thermal conductivity material under a high heat flux. Under such conditions gasification occurs in such a narrow temperature window as to be considered as effectively occurring at a specific temperature. However, it is important to realise that a sacrificial additive also encompasses any material that decomposes endothermically on heating- so-called heat-sink additives. An obvious candidate here is a hydrated mineral filler, such as alumina trihydrate or magnesium hydroxide, which liberates water on heating, consuming energy in the process. The dynamics of endothermic thermal degradation of hydrated materials specifically have been investigated previously in [22] and so in this contribution the general behaviour of a heat-sink additive is discussed.

**Figure 1:**
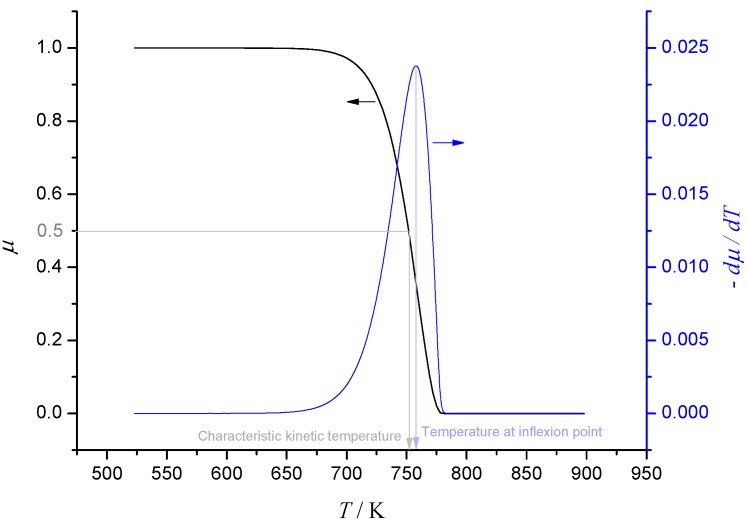
Single-step first-order Arrhenius kinetics at constant heating rate.

Consumption of a reactant during thermal degradation is often modelled using Arrhenius kinetics. Assuming first-order kinetics for simplicity, suppose that the ratio of mass to initial mass μ of a reactant is given by *d*μ/*dt* = −*A*exp(−*T_A_*/*T*)μ, where *A*, *T_A_* are the pre-exponential factor and activation temperature respectively. When heating rate *H* = *dT*/*dt* is constant, the graph of μ *vs.* *T* has a characteristic shape shown in Figure 1, with a single inflexion point located at the solution of *T_A_*/*T*^2^ = *A*exp(−*T_A_*/*T*)/*H*. Putting *z* = *T_A_*/*T*, *J* = ln(*AT_A_*/*H*), this last equation becomes *z*^2^ = *e^J−Z^* and it may be shown that an acceptable approximate solution is given by

(9) $$z\approx \frac{J}{J+2}\left\{2\left(1-\ln J\right)+J\right\},$$

which has error less than 2% when *J* > 11.

Clearly the inflexion point provides a characteristic temperature at which the degradation reaction proceeds. However a more intuitive, but equally valid, characteristic temperature is the temperature at which half of the reactant has been consumed. This is defined as the *characteristic kinetic temperature (CKT)*, which will be denoted by *T*_1/2_ [22,23]. The temperature at inflexion and *T*_1/2_ are shown in Figure 1 and it transpires that these are always close together. In fact, it may be shown that if we set *z*_1/2_ = *T_A_*/*T*_1/2_, then to a good degree of approximation,

(10) $$z_{1/2}\approx \left(J+0.3665\right)\left(1-\frac{2\ln\left(J+0.3665\right)}{J+2.8253}\right).$$

**Figure 2:**
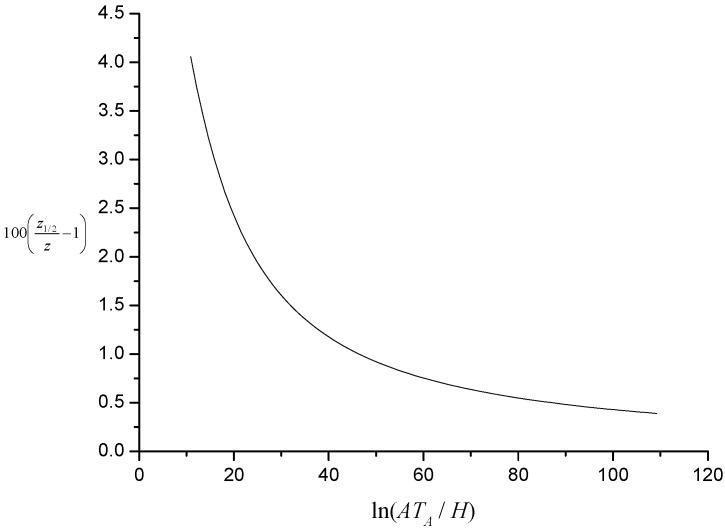
Illustration of percentage relative difference between characteristic kinetic temperature and temperature at inflexion.

The percentage relative difference between *z* and *z*_1/2_, *i.e.*, 100(*z*_1/2_ − *z*)/*z*, is shown plotted as a function of *J* in Figure 2. Note that when *J* is large, *z*_1/2_ ~ *J*.

Another interesting feature of the CKT is that it has only weak dependence on the heating rate *H*. To see this, consider the definition of *J* = ln(*AT_A_*/*H*) = ln(*AT_A_*) − ln*H*. Now given realistic values for *A* and *T_A_*, it transpires that unless *H* is very large indeed (or very small) then *|*ln*H*| << ln(*AT_A_*). This observation has considerable importance when one analyses the influence of heat-sink additives on thermal protection, as will become apparent below.

Now consider a reactive material *P* that endothermically degrades on heating in a first-order process to give a product P*, then assuming for convenience that the thermal properties of *P* and P* are the same, the energy equation for *P* will be ∂*T*/∂*t* = α∂^2^*T*/∂*y*^2^ + (∆*H*/*c*)*d*μ/*dt*. Here, ∆*H* is the heat of the degradation reaction and μ is the mass fraction of *P* remaining. The degradation reaction is assumed to proceed according to *d*μ/*dt* = −*k*μ, as above. If we set τ = α*t*/*l*^2^ (as above), θ*_k_* = 1/ln(*l*^2^*A*/α), *x* = *y*/*l*, θ = *T*/*T_A_*, ε = ∆*H*/*cT_A_*, then the energy and rate equations in dimensionless terms are

(11) $$\frac{\partial \theta }{\partial \tau }=\frac{\partial^{2}\theta }{\partial x^{2}}-\varepsilon \mu \exp\left(\frac{1}{\theta_{k}}-\frac{1}{\theta }\right),\frac{\partial \mu }{\partial \tau }=-\mu \exp\left(\frac{1}{\theta_{k}}-\frac{1}{\theta }\right).$$

Here θ*_k_* plays the role of the CKT discussed above and ε is the ratio of reaction heat to sensible heat.

So that we may concentrate on the effect of the heat sink reaction, suppose that the exposed surface (at *y* = *l*) is subject to a constant heat flux *q"* and the unexposed surface (*y* = 0) is well insulated, so that the boundary conditions are ∂θ/∂*x* = G at *x* = 1 and ∂θ/∂*x* = 0 at *x* = 0, where *G* = *lq"*/(*λT_A_*).

**Figure 3:**
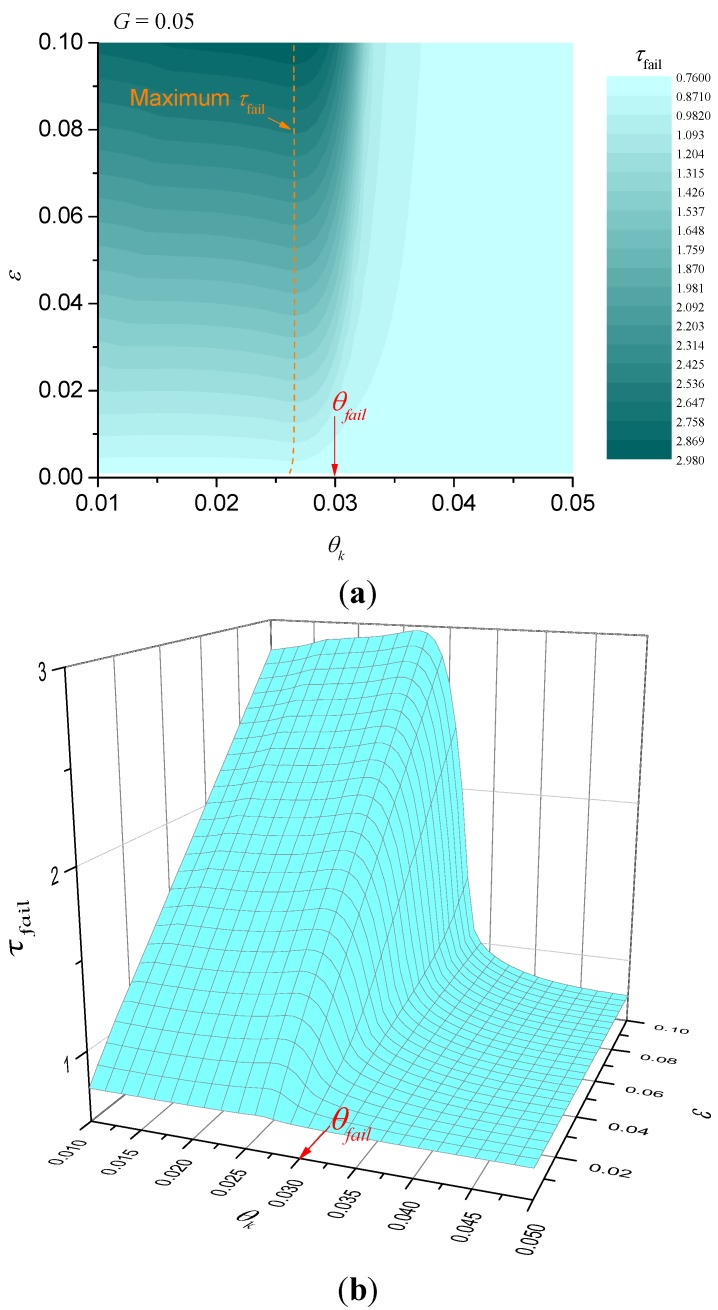
Illustration of dimensionless failure time for *G* = 0.05 (failure at unexposed surface). (**a**) contour plot; (**b**) surface plot.

**Figure 4:**
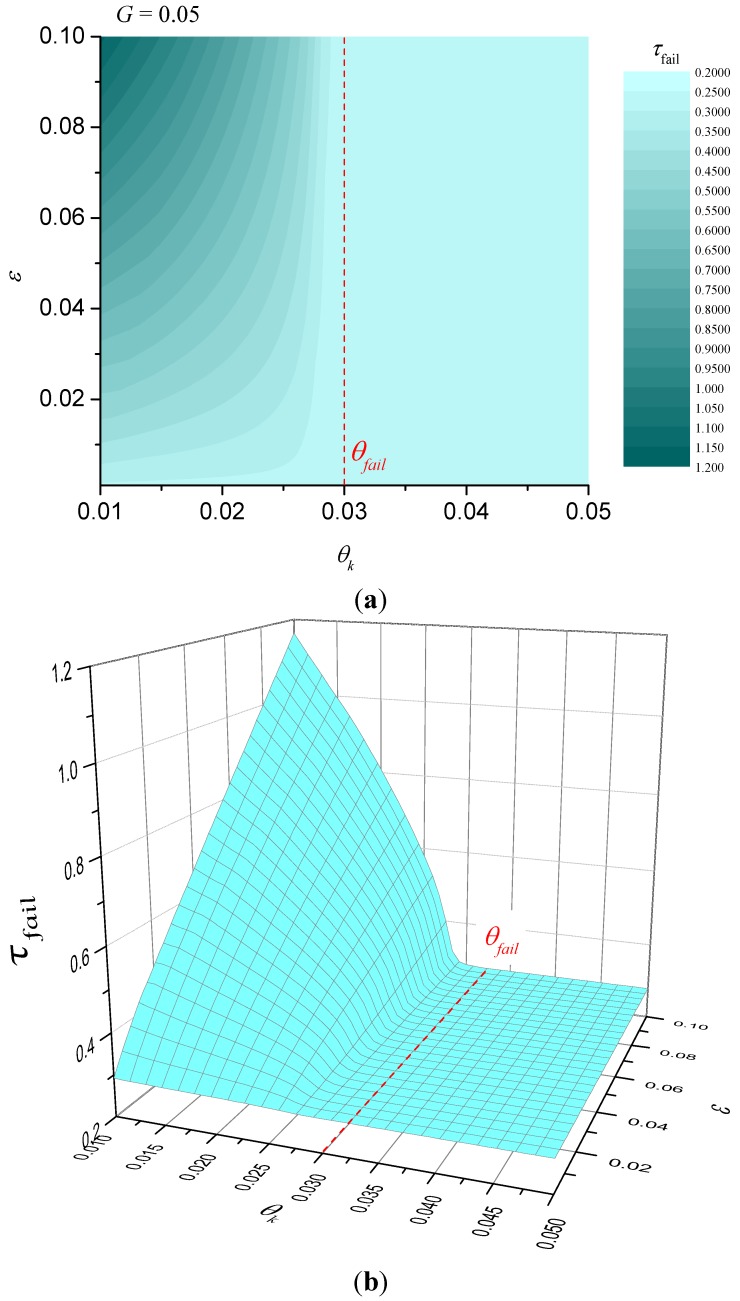
Illustration of dimensionless failure time for *G* = 0.05 (failure at exposed surface). (**a**) contour plot; (**b**) surface plot.

When the heat-sink material is being used to thermally protect a substrate, we are interested in the time taken for the temperature at the unexposed face to reach a critical value. Let the critical temperature be *T_fail_* and define θ*_fail_* = *T_fail_*/*T_A_*. Numerical solutions for the dimensionless time taken until failure are plotted in Figure 3. Alternatively, if we are interested in ignition resistance, then the time taken for the exposed surface to reach a critical temperature and numerical solutions for this case are shown in Figure 4. The detailed solution behaviour has been investigated numerically for other values of dimensionless heat flux *G* in the range 0.001 ≤ *G* ≤ 0.1 and the qualitative behaviour is unchanged from that shown in Figure 3 and Figure 4.

Note that in general, for fixed θ*_fail_*, the failure time will be such that

(12) $$t_{fail}=\frac{l^{2}}{\alpha }f\left(\theta_{k},\varepsilon ,G\right).$$

It is obvious that $\tau_{fail}$ is a decreasing function of *G* indicating that for fixed heat flux the parameter λ*T_A_*/*l* should be as small as possible. Numerical calculations similar to the parameter maps of Figure 3 and Figure 4 were performed to confirm this, but have not been included for brevity. For the case of failure at the unexposed face, Figure 3 shows that the best performance (greatest failure time) occurs when θ*_k_* < θ*_fail_* and when ε is as large as possible. However, for fixed ε there is clearly an optimum value for θ*_k_* (shown in Figure 3 as a dashed curve) that gives the best performance, but τ*_fail_* is only a weak function of θ*_k_* at values below the optimum. In other words, for fixed ε, provided that the characteristic kinetic temperature θ*_k_* is sufficiently below θ*_fail_*, it does not matter how much less θ*_k_* is than θ*_fail_*. The behaviour for failure at the exposed surface is somewhat different, however. Figure 4 indicates that τ*_fail_* is an increasingly strong function of θ*_k_* as ε increases. In fact, there are clear benefits in trying to make the characteristic kinetic temperature as small as possible for a given ε.

**Figure 5:**
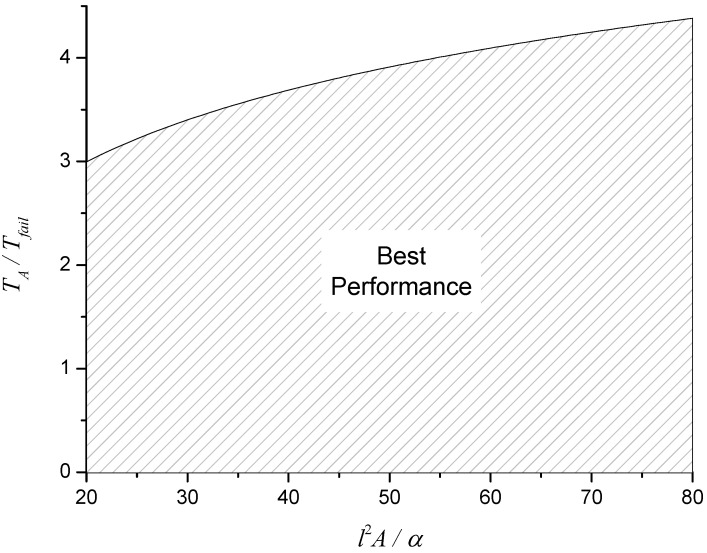
Region of best performance for heat sink materials.

For both cases, there is a clear requirement that

(13) $$\frac{\ln\left(l^{2}A/\alpha \right)}{T_{A}}>\frac{1}{T_{fail}},$$

which is illustrated in Figure 5. This last relation combined with the observation above regarding λ*T_A_*/*l* suggests that the best performance for fixed heat flux is obtained when:

■ λ and *T_A_* are small,
■ *l*, ρ and *A* are large,
■ ∆*H* is large.

**Figure 6:**
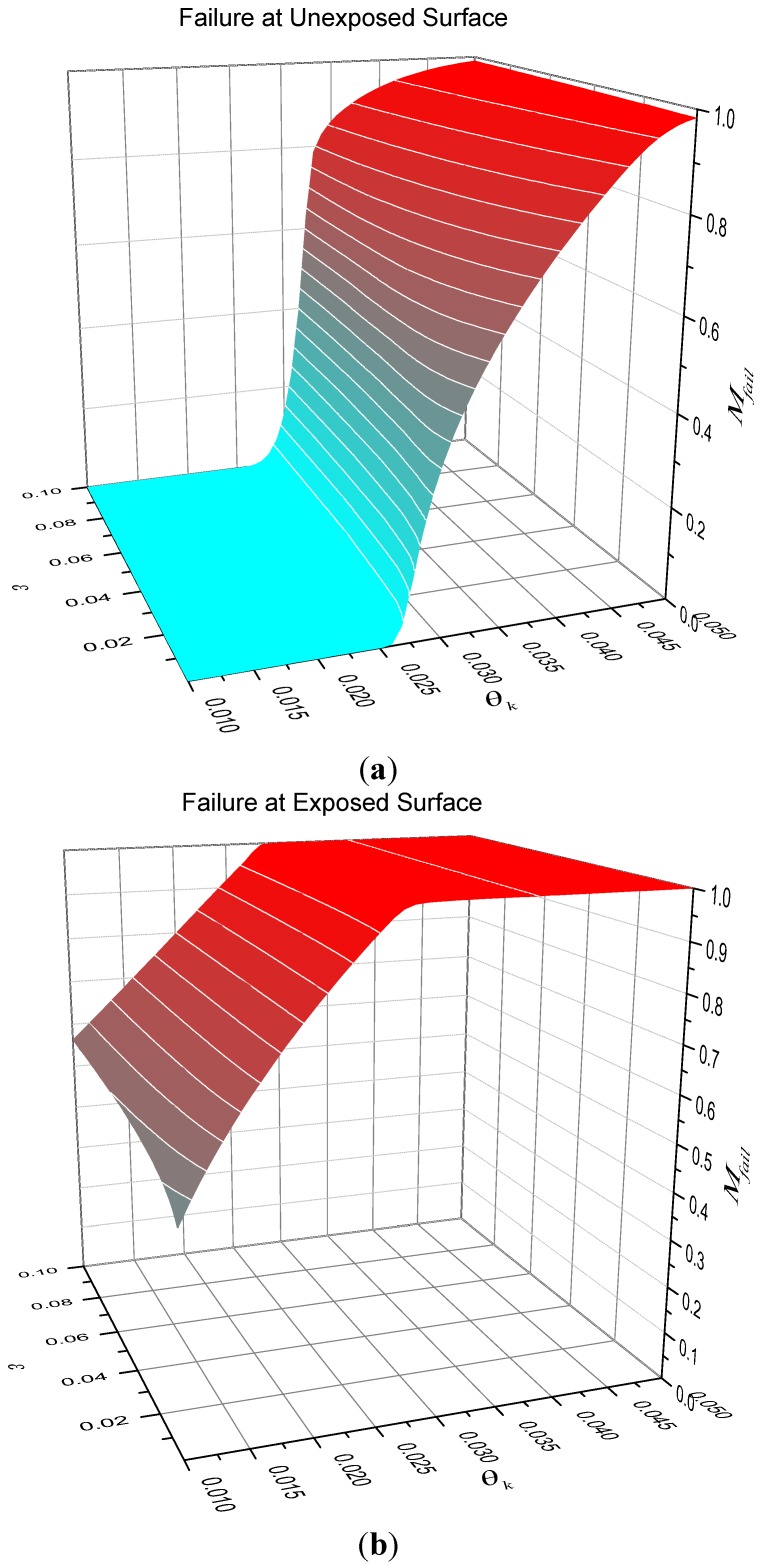
Mass fraction of heat sink at failure at *G* = 0.05. (**a**) contour plot; (**b**) surface plot.

Figure 6 shows surface plots of the mass fraction of reactant throughout the entire solid at failure $M_{fail}=\int_{0}^{l}\mu (x,\tau_{fail})dx$ for the two cases when failure occurs at the unexposed face (top) and exposed face (bottom). It is apparent from this figure that the poorest performance for thermal protection of a substrate occurs when little of the heat sink additive is consumed before failure occurs. For the case of failure at the exposed surface, failure time increases approximately in proportion to the amount of heat sink additive consumed.

## 4. Intumescent Chars

Another successful strategy for thermal protection of a vulnerable substrate is the production of a highly porous foamed char. The desirable thermal properties of an insulating layer have already been discussed above and detailed models of specific intumescent systems have already been published [6,24,25,26]. Analysis of such systems is a complex affair as there are many different chemical and physical processes occurring simultaneously that interact to produce the final thermal characteristics. However the advantageous features of a general system have not been explored in detail in any of these contributions and as we have already explored the important parameters for a static insulation layer above, in this section we concentrate on the heat transfer characteristics of an expanding layer.

Consider an insulating layer that is expanding at a prescribed rate with a fixed heat flux applied at one surface. We are interested in the insulating effect of the expanding layer as time progresses and in particular the time taken for the temperature at the unexposed face to reach a prescribed value (the failure time). For simplicity, the thermal properties of the expanding layer will be taken to be constant so that the dynamics of the expansion process can be analysed without confusing additional effects. We take as a model system a reactive layer that produces a foamed char on heating. The thermal properties of the char are fixed as soon as it is produced and its thickness increases steadily from zero with increasing time.

**Figure 7:**
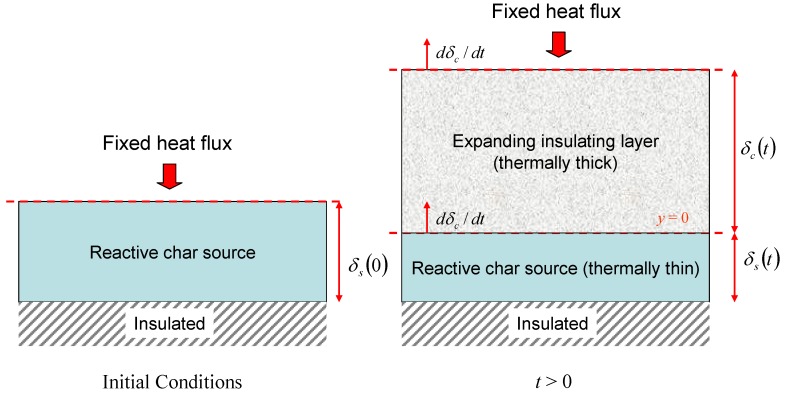
Illustration of simple expanding char model.

Let the char have thickness δ*_c_*(*t*), thermal diffusivity α*_c_* and occupy the region 0 ≤ *y* ≤ δ*_c_*(*t*). It is a straightforward matter to verify that the temperature in the expanding layer is given by the solution of $\partial T/\partial t=-{\dot{\delta }}_{c}\partial T/\partial y+\alpha_{c}\partial^{2}T/\partial y^{2}$, where ${\dot{\delta }}_{c}=d\delta_{c}/dt$ is the expansion rate. Suppose that the reactive layer producing the char (the char source) has thickness δ*_s_* and density ρ*_s_*. As char is produced the char source is consumed, so that $-\rho_{s}{\dot{\delta }}_{s}=\rho_{c}{\dot{\delta }}_{c}$. The quantity *E* = ρ*_s_*/ρ*_c_* is the expansion ratio for the char and hence the ultimate char thickness will be given by δ_∞_ = *E*δ_0_, where δ_0_ = δ*_s_*(0) is the initial thickness of the reactive layer. The expression for the ultimate expanded char thickness comes about from the fact that in this simple model it is assumed that all of the reactive layer is converted into char. Hence conservation of mass implies that, for constant cross-sectional area, $\delta_{s}\rho_{s}=\delta_{\infty }\rho_{c}\Rightarrow \delta_{\infty }/\delta_{s}=\rho_{s}/\rho_{c}=E$. Again for simplicity we shall take the heat flux on the exposed surface of the expanding layer *q"* to be constant and the unexposed face of the char source to be adiabatic so that effect of the char expansion can be explored without the additional effects of heat losses. These features are summarised in Figure 7.

We assume that the char source is sufficiently thin so that its temperature *T_s_* is uniform and conservation of energy implies that when *t* > 0, $\rho_{s}cd\left(\delta_{s}T_{s}\right)/dt=\lambda_{c}\partial T/\partial y{|}_{y=0^{+}}+\rho_{s}cT_{s}{\dot{\delta }}_{s}$. Here $\lambda_{c}\partial T/\partial y{|}_{y=0^{+}}$ is the heat flux in the char layer immediately above the char source, λ*_c_* is the thermal conductivity of the char and *c* is the specific heat capacity of char source (assumed to be the same as the char). Thus the temperature of the char source is given by the solution of

(14) $$\frac{dT_{s}}{dt}=\left\{ \begin{array}{l} \frac{{\dot{q}}^{\prime \prime }}{\delta_{0}\rho_{s}c},t=0,\\ \frac{\lambda_{c}}{\delta_{s}\rho_{s}c}{\frac{\partial T}{\partial y}|}_{y=0^{+}},t>0. \end{array}\right.$$

Now, let *t_c_* be the time taken for the char to expand to its ultimate thickness, so that

(15) $$\delta_{c}\left(t\right)=\left\{ \begin{array}{l} {\dot{\delta }}_{c}t,0\leq t\leq t_{c},\\ \delta_{\infty },t>t_{c} \end{array}\right.$$

and set $x=y/\delta_{c}\left(t\right)$, $\tau =t/t_{c}$, $\theta =T/T_{a}$, $\varepsilon =\alpha_{c}/\left(\delta_{\infty }{\dot{\delta }}_{c}\right)$, $\beta =\delta_{\infty }{\dot{q}}^{\prime \prime }/\left(\lambda_{c}T_{a}\right)$. It is now apparent that the behaviour of the simple expanding char model is determined by two parameters (ε and β) and physically, ε corresponds to the ratio of heat diffusion rate to expansion rate and β corresponds to the ratio of external heat flux to diffusion heat flux. In these variables it may be shown that the simple expanding char problem may be written as

(16) $$\frac{\partial \theta }{\partial \tau }=\left\{ \begin{array}{l} \left(\frac{x-1}{\tau }\right)\frac{\partial \theta }{\partial x}+\frac{\varepsilon }{\tau^{2}}\frac{\partial^{2}\theta }{\partial x^{2}},0<\tau \leq 1,\\ \varepsilon \frac{\partial^{2}\theta }{\partial x^{2}},\tau >1, \end{array}\right.$$

with boundary conditions

(17) $${\frac{\partial \theta }{\partial x}|}_{x=0^{+}}=\left\{ \begin{array}{l} \frac{\tau \left(1-\tau \right)}{\varepsilon }{\frac{\partial \theta }{\partial \tau }|}_{x=0^{-}},0<\tau \leq 1,\\ 0,\tau >1, \end{array}\right.$$

(18) $${\frac{\partial \theta }{\partial x}|}_{x=1}=\left\{ \begin{array}{l} \beta \tau ,0<\tau \leq 1,\\ \beta ,\tau >1 \end{array}\right.$$

and initial condition θ(*x*, 0) = 1. When τ is small it is necessary to seek a series expansion for the solution because of the singularities in Equation (16) and this shows that the initial behaviour is given by:

(19) $$\theta \sim 1+\beta \tau \left(\varepsilon +x\right)+O\left(\tau^{2}\right)$$

When the expansion rate is large, the char layer becomes fully developed long before the failure temperature is reached and so we would expect the failure time for this case to be dominated by the solution of the equivalent heat transfer problem in a layer of constant thickness (the *static solution*). For this case, we simply need solutions of ∂θ/∂τ = ε∂^2^θ/∂*x*^2^, 0 ≤ *x* ≤ 1, with ∂θ/∂*x* = 0 on *x* = 0 and ∂θ/∂*x* = β on *x* = 1. It is a straightforward matter to construct the solution of the static problem, giving the temperature on the unexposed face as

(20) $$\theta_{s}^{\left(static\right)}=\beta \varepsilon \tau +1-\frac{\beta }{6}-\frac{2\beta }{\pi^{2}}\sum_{n=1}^{\infty }\frac{{\left(-1\right)}^{n}}{n^{2}}e^{-n^{2}\pi^{2}\varepsilon \tau }$$

From this, we calculate the time taken $\tau_{\mathrm{fail}}^{\left(\mathrm{static}\right)}$ for $\theta_{s}^{\left(\mathrm{static}\right)}$ to reach the failure temperature θ*_fail_*. It is apparent from above that ${\varepsilon \tau }_{\mathrm{fail}}^{\left(\mathrm{static}\right)}$ = *f*(θ*_fail_*, β) and numerical results show that a good approximation for *f* is given by the function exp(*c*_0_ + *c*_1_*z* + *c*_2_*z*^2^ + *c*_3_*z*^3^), where *z* = lnβ. For example, when θ*_fail_* = 2.5 the coefficients *c_j_* are given by *c*_0_ = 0.5155, *c*_1_ = −0.9531, *c*_2_ = 0.1072, *c*_3_ = −0.0053.

When the expansion rate is small but the heating rate is high, the failure time will be small and we would expect the temperature distribution through the slowly expanding char to be close to quadratic in *x*. Under these pseudo-steady conditions it seems reasonable to seek an approximate solution of the form θ(*x*, τ) ~ θ_0_(τ) + *x*θ_1_(τ) + *x*^2^θ_2_(τ) and the failure time will be given by the solution of θ*_fail_* = θ_0_(τ*_fail_*). Substitution into the model equations eventually yields

(21) $$\theta_{0}\left(\tau \right)=\left\{ \begin{array}{l} 1+\frac{\varepsilon^{2}\beta }{2\omega }\ln\left\{\left(\frac{\omega +1/2}{\omega -1/2}\right)\left(\frac{\omega +\tau -1/2}{\omega -\tau +1/2}\right)\right\},0\leq \tau \leq 1,\\ 1+\frac{\varepsilon^{2}\beta }{\omega }\ln\left(\frac{\omega +1/2}{\omega -1/2}\right)+\varepsilon \beta \left(\tau -1\right),\tau >1, \end{array}\right.$$

where $\omega =\sqrt{\varepsilon +1/4}$. Hence the failure time $\tau_{fail}^{\left(ps\right)}$ will be given by

(22) $$\tau_{fail}^{\left(ps\right)}=\left\{ \begin{array}{l} \frac{\varepsilon \left(e^{2z}-1\right)}{\omega +1/2+\left(\omega -1/2\right)e^{2z}},z<\ln\left(\frac{\omega +1/2}{\omega -1/2}\right),\\ 1+\frac{\varepsilon }{\omega }\left\{z-\ln\left(\frac{\omega +1/2}{\omega -1/2}\right)\right\},z\geq \ln\left(\frac{\omega +1/2}{\omega -1/2}\right), \end{array}\right.$$

where *z* = ω(θ*_fail_* − 1)ε^2^β.

**Figure 8:**
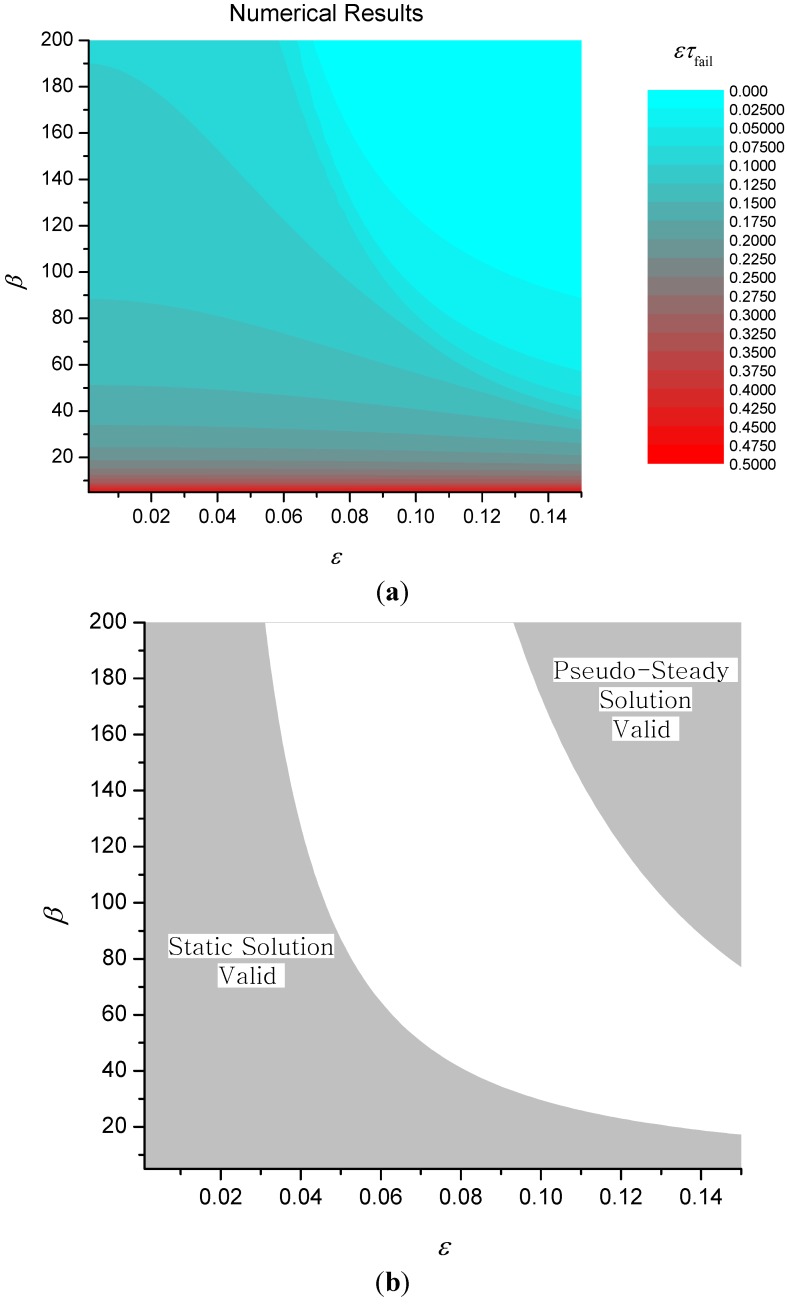
Contours of ετ_fail_ (**a**) and validity of approximate solutions for simple expanding char model (**b**).

Numerical results for ετ*_fail_* (with θ*_fail_* = 2.5) are shown in Figure 8 together with regions where the two approximate solutions given by Equations (20) and (22) above are valid. An approximate solution is deemed *valid* in this sense when the percentage relative error between the approximate and numerical solutions is less than 5%.

It transpires that the solution to the static problem also has another important physical interpretation. Since the thickness of the expanding layer is always less than or equal to the ultimate thickness, it follows that the conduction heat transfer coefficient λ*_c_*/δ*_c_* is always greater than λ*_c_*/δ_∞_. Now from Equation (1), this implies that the failure time for the expanding char will always be less than the failure time for the static solution, *i.e.*, τ*_fail_* < $\tau_{fail}^{\left(static\right)}$. In other words, the failure time for the static solution represents the best possible performance and so it can be used as a benchmark to determine the effectiveness of the expanding char. Hence we can define a theoretical efficiency η given by

(23) $$\eta \left(\varepsilon ,\beta \right)=\frac{\tau_{fail}}{\tau_{fail}^{static}}.$$

**Figure 9:**
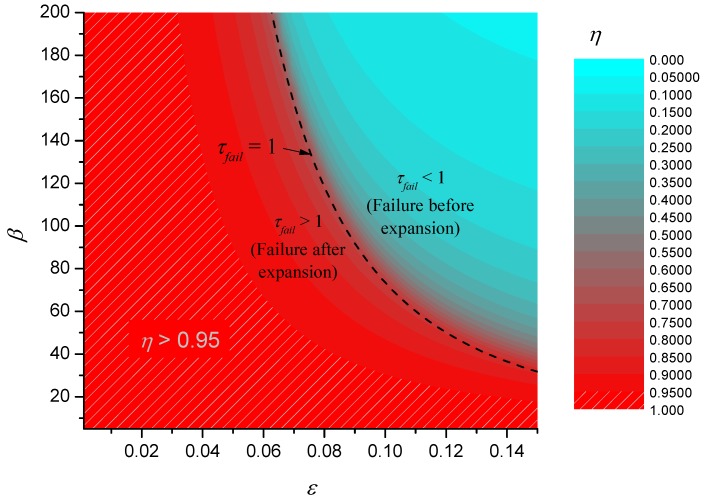
Efficiency of simple expanding char model.

Figure 9 shows a contour plot of the theoretical efficiency together with the contour C_1_ corresponding to τ*_fail_* = 1. This contour is interesting because it corresponds to the case when the failure temperature is reached just at the point that the char finishes expanding. If τ*_fail_* < 1, (the region above and to the right of C_1_) then this implies that the failure temperature has been reached before expansion is complete. If τ*_fail_* > 1 (the region below and to the left of C_1_) then failure occurs after complete expansion. In fact, C_1_ closely corresponds to a watershed that divides a basin for ετ*_fail_* where performance is poor from a region of good performance.

Analysis of the numerical results shows that the equation of C_1_ is given to a good degree of approximation by β = 2.604 + 0.453/ε^2.195^. Multiplying this expression by ε, we have that the region where failure occurs before complete expansion is given by εβ > 2.604ε + 0.453/ε^1.195^ and as ε varies, it is obvious that the right-hand side of this inequality is always greater than or equal to 2.338. Hence. it follows that if ${\dot{\delta }}_{c}\rho_{c}c>0.428{\dot{q}}^{\prime \prime }/T_{a}$, then the expansion rate will *always* be sufficiently large to ensure that failure occurs after complete expansion, for *any* value of char thermal conductivity.

The contour plot of η also shows that there is a large region of parameter space where efficiency is close to 1. Note that given the definition of η, the hatched region of Figure 9 and the region of validity of the static solution in Figure 8 are identical, implying that to a good degree of approximation, the failure time in the region η > 0.95 will be given approximately by the static solution.

**Figure 10:**
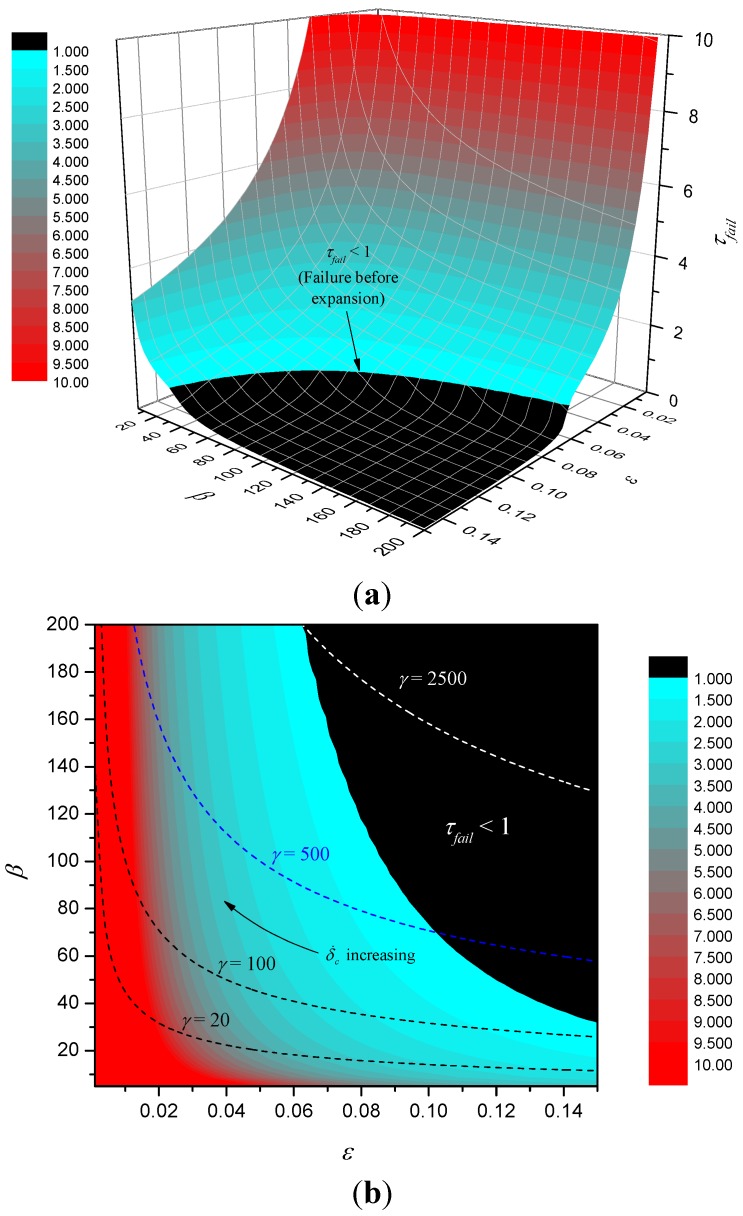
Surface map of τ*_fail_* (**a**) and contour plot (**b**)*.*

Figure 10 shows a surface map and corresponding contour map of τ*_fail_*. The figure shows that in the basin where failure occurs before complete expansion failure time is generally small, but once out of the basin, τ*_fail_* increases rapidly with reducing ε and reducing β. Given the definitions of ε and β, we can write $\varepsilon =\alpha_{c}/{\dot{\delta }}_{c}^{2}t_{c}$, $\beta ={\dot{\delta }}_{c}t_{c}{\dot{q}}^{\prime \prime }/\left(\lambda_{c}T_{a}\right)$. Hence, for fixed *t_c_*, expanded char thermal properties and heat flux *q"*, it follows that as expansion rate ${\dot{\delta }}_{c}$ varies, ε and β are related through β = (γ/ε)^1/2^, where $\gamma =t_{c}{{\dot{q}}^{\prime \prime }}^{2}/\lambda_{c}\rho_{c}cT_{a}^{2}$ and these curves are plotted for various values of γ in the contour plot. As expansion rate increases we proceed from right to left along the curve. Note that as γ increases, larger portions of the curves lie in the basin of small failure time.

**Figure 11:**
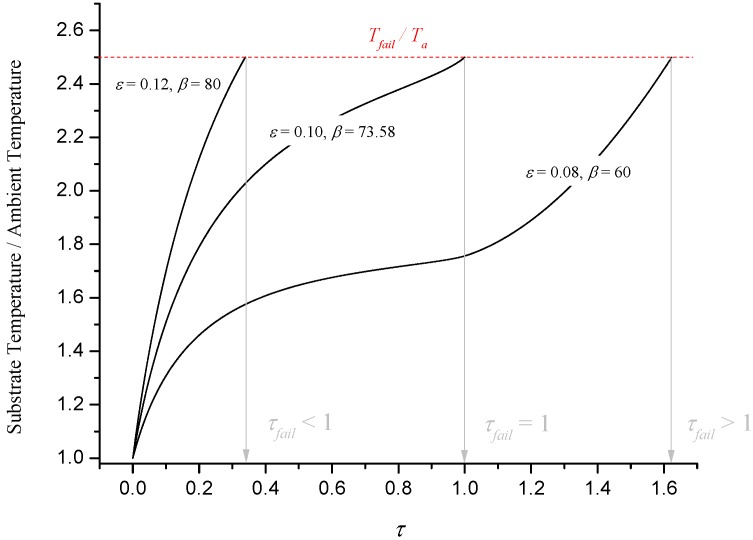
Illustration of numerical results for substrate temperature.

Figure 11 illustrates numerical solutions for dimensionless substrate temperature for three cases: a point where failure occurs before expansion is complete (ε = 0.12, β = 80), a point on C_1_ where failure occurs just as expansion is complete (ε = 0.10, β = 73.58) and a point where failure occurs after expansion (ε = 0.08, β = 60).

## 5. Conclusions

Pore shape and distribution is critical in optimising the thermal performance of inert insulating layers. For most practical applications, pore size is not sufficiently large for internal convection to occur, however as temperature increases radiation heat transfer becomes important. For small pores it is possible to show that radiation heat transfer rate increases with pore size, suggesting that pore size should be kept as small as possible. If the goal of the thermal protection is maximise failure time of a substrate, it transpires that λ*_tot_*/(1 − φ) should be as small as possible, where λ*_tot_* is the total effective thermal conductivity including radiation augmentation. Alternatively if the goal is ignition resistance, then the analysis above shows that λ*_tot_*/(1 − φ) should be as large as possible.

For a simple reactive heat-sink material (RHSM) it transpires that the best thermal performance occurs when *T_A_*/ln(*Da*) < *T_fail_*, where *T_A_* is the activation temperature for the endothermic reaction and *Da* is the Damköhler number *l*^2^*A*/α (here *l* is the thickness of the RHSM, *A* is the pre-exponential factor and α is thermal diffusivity). Furthermore, failure time is an approximately linearly increasing function of the Stefan parameter ∆*H*/*cT_A_*, where ∆*H* is the heat absorbed per kg of RHSM and *c* is specific heat capacity.

For the simple expanding char model, it was shown that dimensionless failure time is a function of two dimensionless parameters: *t_fail_*/*t_c_* = *f*(ε, β), where $\varepsilon =\alpha_{c}/{\dot{\delta }}_{c}^{2}t_{c}$, $\beta ={\dot{\delta }}_{c}t_{c}{\dot{q}}^{\prime \prime }/\lambda_{c}T_{a}$. Here αc, λc are the thermal diffusivity and thermal conductivity of the expanded char respectively, tc is the time taken for expansion and ${\dot{\delta }}_{c}$ is the expansion rate. Numerical results indicate that there is a basin where dimensionless failure time is low, given approximately by β > 2.604 + 0.453/ε^2.195^ for the case of a maximum substrate temperature (in K) of 2.5*T_a_*. Inside the basin char expansion is slow enough such that failure occurs before expansion is complete and outside the dimensionless failure time increases rapidly as both ε and β reduce. Furthermore if εβ < 2.338, or in terms of the original variables ${\dot{\delta }}_{c}\rho_{c}c>0.428{\dot{q}}^{\prime \prime }/T_{a}$, then the expansion rate will be sufficiently large to ensure that failure occurs after complete expansion no matter what the value of the char thermal conductivity λ*_c_*.

In both cases where thermal protection is afforded by a reactive component (either a heat sink reaction or an expanding insulation layer) the numerical results demonstrate clearly the seemingly obvious but important requirement that, for prescribed heating conditions, the reaction must proceed to completion for the best possible performance. In practice, this means that for effective thermal protection, the kinetics of the reaction must be matched to the heating rate imposed by the external source.
